# Therapeutic importance of synthetic thiophene

**DOI:** 10.1186/s13065-018-0511-5

**Published:** 2018-12-19

**Authors:** Rashmi Shah, Prabhakar Kumar Verma

**Affiliations:** Department of Pharmaceutical Sciences, Maharshi Dayanand University, Rohtak, Haryana 124001 India

**Keywords:** Thiophene, Heterocyclic compounds, Combinatorial library, Antimicrobial

## Abstract

Thiophene and its substituted derivatives are very important class of heterocyclic compounds which shows interesting applications in the field of medicinal chemistry. It has made an indispensable anchor for medicinal chemists to produce combinatorial library and carry out exhaustive efforts in the search of lead molecules. It has been reported to possess a wide range of therapeutic properties with diverse applications in medicinal chemistry and material science, attracting great interest in industry as well as academia. It has been proven to be effectual drugs in present respective disease scenario. They are remarkably effective compounds both with respect to their biological and physiological functions such as anti-inflammatory, anti-psychotic, anti-arrhythmic, anti-anxiety, anti-fungal, antioxidant, estrogen receptor modulating, anti-mitotic, anti-microbial, kinases inhibiting and anti-cancer. Thus the synthesis and characterization of novel thiophene moieties with wider therapeutic activity is a topic of interest for the medicinal chemist to synthesize and investigate new structural prototypes with more effective pharmacological activity. However, several commercially available drugs such as Tipepidine, Tiquizium Bromides, Timepidium Bromide, Dorzolamide, Tioconazole, Citizolam, Sertaconazole Nitrate and Benocyclidine also contain thiophene nucleus. Therefore, it seems to be a requirement to collect recent information in order to understand the current status of the thiophene nucleus in medicinal chemistry research.

## Introduction

As the world’s population is increasing at an alarming rate, health problems have also become a very serious clinical problem. Therefore, it is an urgent requirement for the scientist to design and discover new drug molecules which possibly offers some of the greatest hopes for success in present and future epoch. However, there are still enormous numbers of pharmacologically active heterocyclic compounds which are in regular clinical use [1]. Heterocyclic compounds are extensively distributed in nature and have versatile synthetic applicability and biological activity which helped the medicinal chemist to plan, organize and implement new approaches towards the discovery of novel drugs [2].

**Fig. 1:**
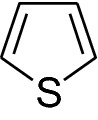
Thiophene

Thiophene (Fig. 1) is a five membered heteroaromatic compound containing a sulfur atom at 1 position. It is considered to be a structural alert with formula C_4_H_4_S, chemical name is thiacyclopentadiene [3].

Thiophene was discovered as a contaminant in benzene [4]. It has the molecular mass of 84.14 g/mol, density is 1.051 g/ml and Melting Point is − 38 °C. It is soluble in most organic solvents like alcohol and ether but insoluble in water. The “electron pairs” on sulfur are significantly delocalized in the π electron system and behaves extremely reactive like benzene derivative. Thiophene forms a azeotrope with ethanol like benzene. The similarity between the physicochemical properties of benzene and thiophene is remarkable. For example, the boiling point of benzene is 81.1 °C and that of thiophene is 84.4 °C (at 760 mmHg) and therefore, both are a well known example of bioisosterism [5]. It can be easily sulfonated, nitrated, halogenated, acylated but cannot be alkylated and oxidized [3].

In medicinal chemistry, thiophene derivatives are very important heterocycles exhibiting remarkable applications in different disciplines. In medicine, thiophene derivatives shows antimicrobial [6], analgesic and anti-inflammatory [7], antihypertensive [8], and antitumor activity [9] while they are also used as inhibitors of corrosion of metals [10] or in the fabrication of light-emitting diodes in material science [11].

### Biological activities of thiophene derivatives

Thiophene nucleus containing compounds show various activities like for example 1-[1-(2,5-dimethylthiophen-3-yl)ethyl]-1-hydroxyurea (**1**) act as an anti-inflammatory agent; the maleate salt of 1-(2,5-dimethylthiophen-3-yl)-3-(5-methyl-1*H*-imidazol-4-yl)propan-1-one (**2**) work as serotonin antagonists and is used in the treatment of Alzheimer’s disease.

**Table:** 

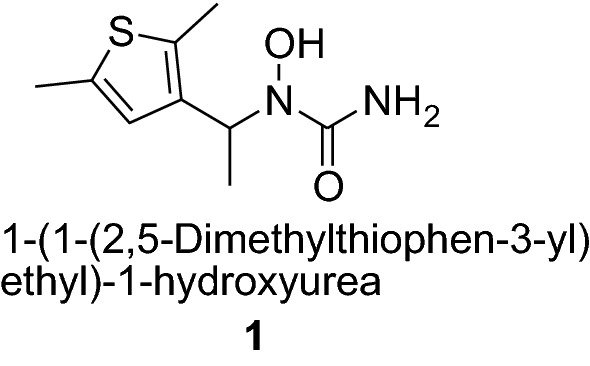	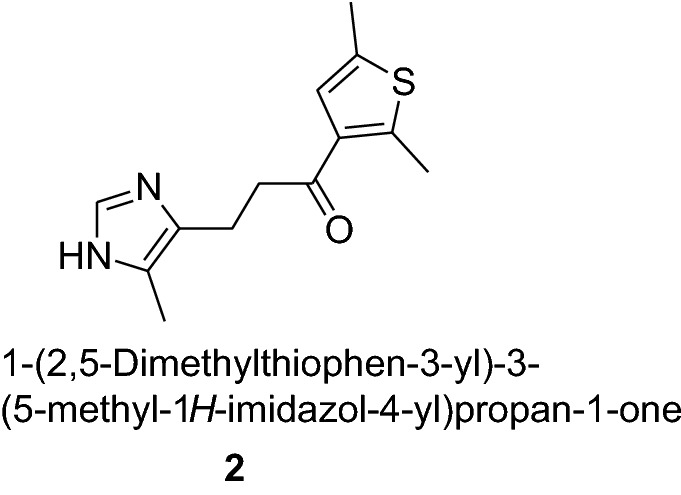

2-Butylthiophene (**3**) is used as a raw material in the synthesis of anticancer agents and 2-octylthiophene (**4**) is used in the synthesis of anti-atherosclerotic agents such as (**5**). It also act as metal complexing agents and in the development of insecticides.

**Table:** 

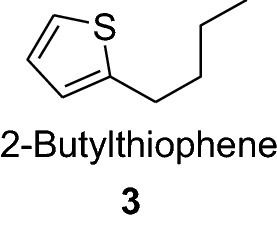	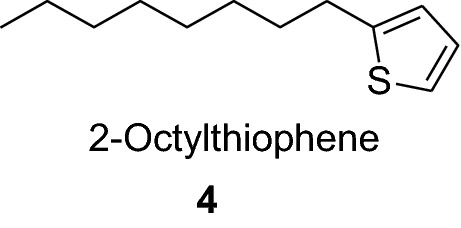

The higher alkylated thiophenes (**6**) has been used extensively as a raw material in patents relating to liquid crystals [12].

**Table:** 

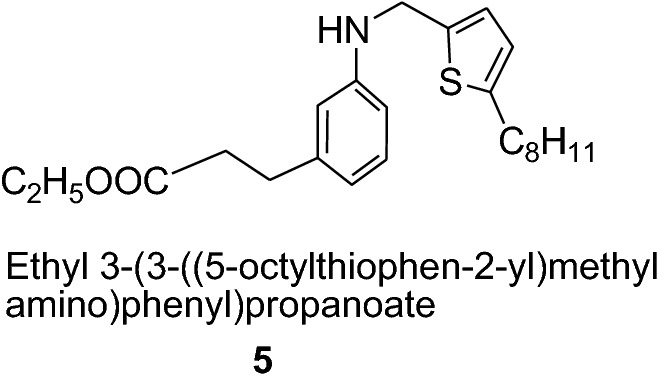	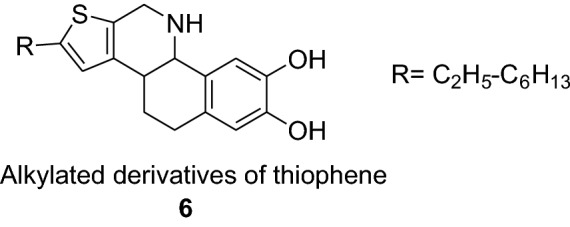

### Antimicrobial activity

Thiophene derivatives show high antimicrobial activity against various microbial infections. Different approaches were made to prove thiophene as antimicrobial agent by different scientist for the discovery of most active thiophene derivatives to the present scenario [13].

**Scheme 1:**
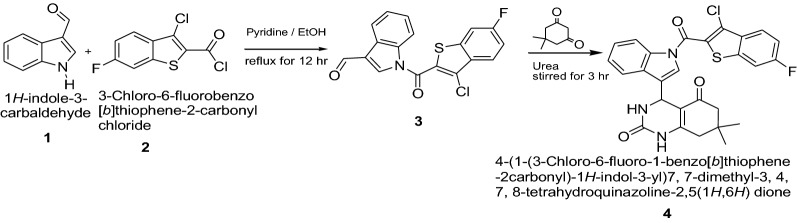
Synthesis of 4-(1-(3-chloro-6-fluoro-1-benzo[*b*]thiophene-2-carbonyl)-1*H*-indol-3-yl)-7,7-dimethyl-3,4,7,8-tetrahydroquinazoline 2,5(1*H*,6*H*)dione

Mehta et al. [14] developed a new class of 4-(1-(3-chlorobenzo[*b*]thiophene-2-carbonyl)-1*H*-indol-3-yl)-7, 7-dimethyl-3,4,7,8-tetrahydroquinazoline 2,5(1*H*,6*H*)dione thiophene derivatives (Scheme 1). These synthesized compounds were screened for their antibacterial activity against three bacterial strains viz. *E. coli, P. aeruginosa, S. aureus* and three fungal strains viz. *C. albicans, A. niger, A. Clavatus* using serial broth dilution method. The standard drug used in this study was ‘Ampicillin’ for evaluating antibacterial activity which showed (50, 100, and 50 μg/ml) MIC against *E. coli, P. aeruginosa* and *S. aureus,* respectively. For antifungal activity ‘Griseofulvin’ was used as a standard drug, which showed (100, 100, and 100 μg/ml) MIC against *C. albicans, A. niger*, and *A. clavatus*, respectively. Among the synthesized derivatives, Compound **4** was found to be good active against *P. aeruginosa.* For the antifungal activity compounds **4** was considered as good active against *A. niger* and *A. clavatus.* The results of synthesized compounds presented in Table 1.

**Table 1:** Biological activity of synthesized compounds

S. no.	Antibacterial strains				Antifungal strains	
	Gram negative		Gram positive			
	*E. coli*	*P. aeruginosa*	*S. Aureus*	*C. albicans*	*A. niger*	*A. clavatus*
**4**	500	100	250	250	100	100
SD	100	100	50	100	100	100

Minimum inhibitory concentrations was expressed as (µg/ml)

SD =  Ampicillin for antibacterial drug; SD = Griseofulvin for antifungal drug

**Scheme 2:**
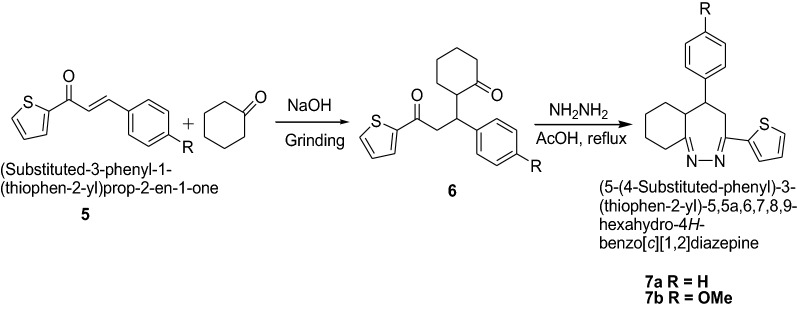
Synthesis of diazepines (**7a**, **7b**)

Mazimba [15] synthesized thiophene analogues of chalcones in good yields by condensation of 2-acetylthiophene and salicylaldehydes using Scheme 2. 1,5-Diketones were formed by solvent-free michael addition of cyclohexanone and 2-thienylchalcones devoid of hydroxyl groups which were used as synthons for synthesis of diazepines. The synthesized compounds were screened for in vitro antimicrobial activities against *S. aureus*, *E. coli*, *B. subtilis*, *P. Aeruginosa* and *C. Albicans* using dilution method. The compounds were found to show moderate to good antibacterial and antifungal activities. Among the tested compounds, diazepines (**7a**, **b**) exhibited excellent antibacterial (*S. aureus* and *P. aeruginosa*) and antifungal (*C. albicans*) activities. The results showed the importance of the carbon–nitrogen bond in biological systems because of which antimicrobial activities for these N-containing compounds were reported. The results of synthesized compounds showed in Table 2.

**Table 2:** Antimicrobial activity of thiophen-2-yl-chalcone derived heterocyclic compounds

S. no.	*S. aureus*	*E. coli*	*B. subtilis*	*P. aeruginosa*	*C. albicans*
**7a**	0.313	0.625	0.625	0.313	0.313
**7b**	0.313	0.625	0.625	0.313	0.313
Ciprofloxacin	0.625	0.625	0.625	0.625	–
Fluconazole	–	–	–	–	0.625

Minimum inhibitory concentration (MIC; µg/ml)

**Scheme 3:**
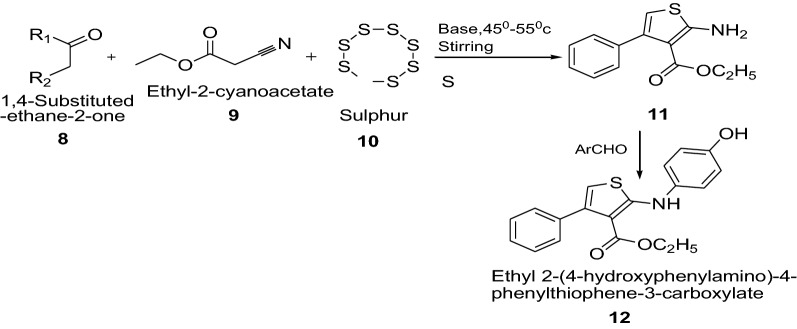
Synthesis of ethyl 2-(4-hydroxyphenylamino)-4-phenylthiophene-3-carboxylate

Prasad et al. [16] synthesized newly ethyl 2-amino-4-phenylthiophene-3-carboxylate derivatives using Scheme 3. The synthesized compounds were screened for their antibacterial activity by using minimum inhibitory concentration (MIC) method by taking ampicillin and streptomycin as standard drug. Among all the synthesized derivatives, compound **12** showed greater inhibitory effect against the organisms used, particularly against *B. subtilis*, *E. coli*, *P. vulgaris* and *S. aureus* with MIC. The present study has given deep insight as the 2-aminothiophene bearing 4-hydroxy benzaldehyde shown significant anti-microbial activity. The compound **12** showed the significant anti-microbial activity among all the synthesized 2-aminothiophene derivatives because of the presence of 4-hydroxy benzaldehyde at second position. The results of synthesized compounds presented in Table 3.

**Table 3:** Antimicrobial activity of 2-aminothiophene derivatives

S. no.	*P. vulgaris*	*B. subtilis*	*E. coli*	*S. aureus*
**12**	25	50	12.5	50
Ampicillin	25	50	12.5	50
Streptomycin	12.5	50	12.5	25

Minimum inhibitory concentration (µg/ml)

**Scheme 4:**
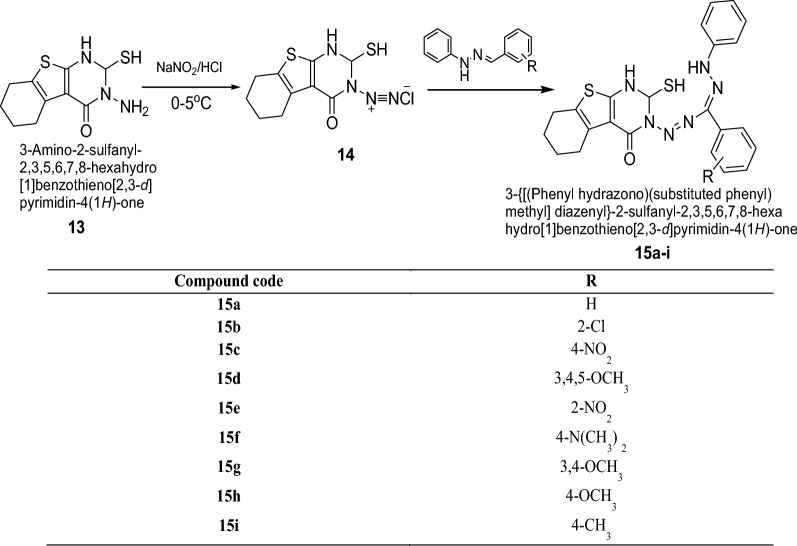
Synthesis of 3-{[(phenylhydrazono)(substitutedphenyl)methyl]diazenyl}-2-sulfanyl-2,3,5,6,7,8-hexahydro [1] benzothieno[2,3-*d*]pyrimidin-4(1*H*)-one (**15a**–**i**)

Lakshmi et al. [17] synthesized 3-{[(phenylhydrazono) (substituted phenyl)methyl]diazenyl}-2-sulfanyl-2,3,5,6,7,8-hexahydro [1] benzothieno[2,3-*d*]pyrimidin-4(1*H*)-one derivatives by using Scheme 4. All the synthesized compounds were screened for their antibacterial and antifungal activities against various microbes such as *B. subtilis*, *E. coli, P. aeruginosa* and *C. albicans* by the cup-plate agar diffusion method. From all the series, compounds **15a**, **15c**, **15g**, **15h**, **15i** were active against *B. subtilis*, compounds **15b**, **15d**, **15e**, **15h**, **15i** were active against *E. coli*, compounds **15a**, **15c**, **15d**, **15e**, **15g**, **15h**, **15i** showed activity against *P. aeruginosa* and compounds **15a**, **15b**, **15c**, **15f**, **15g**, **15h**, **15i** were found active against *C. albicans*. The results of synthesized compounds showed in Table 4.

**Table 4:** Antimicrobial activity of benzothieno[2,3-*c*]chromen-6-one derivatives

Compound	Antibacterial			Antifungal
	*B. subtilis*	*E. coli*	*P. aureginosa*	*C. albicans*
**15a**	11	00	15	10
**15b**	00	10	00	14
**15c**	12	00	12	12
**15d**	00	14	13	00
**15e**	00	10	11	00
**15f**	00	00	00	12
**15** **g**	14	00	14	11
**15** **h**	12	10	12	13
**15i**	14	11	12	12
Ampicillin	15	18	20	–
Fluconazole	–	–	–	15

Minimum inhibitory concentrations was expressed as (µg/ml)

**Scheme 5:**
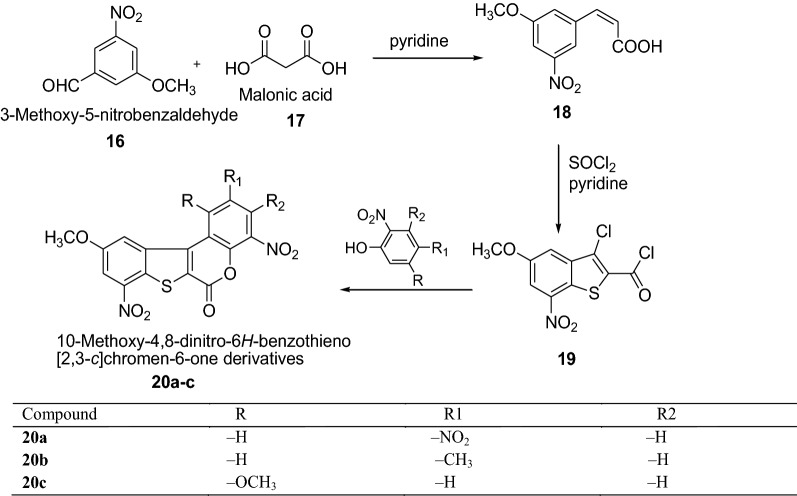
Synthesis of 10-methoxy-4,8-dinitro-6*H*-benzothieno[2,3-*c*]chromen-6-one derivatives (**20a**–**c**)

Havaldar et al. [18] synthesized 10-methoxy-4,8-dinitro-6*H*-benzothieno[2,3-*c*]chromen-6-one derivatives by using Scheme 5. All the synthesized compounds were tested for their antibacterial activity against *S. aureus*, *E. coli*, *B. subtilis* and *S. typhosa* using concentrations of 2 and 5 µg/ml by the ditch plate technique. Among all the series, the compounds **20b** showed a much higher inhibitory effect on the growth of bacteria because of the presence of CH_3_ group. The results of synthesized compounds presented in Table 5.

**Table 5:** Antimicrobial activity of benzothienopyrimidinone derivatives

Compd	*S. aureus*		*E. coli*		*B. Subtilis*		*S. typhosa*	
	2 µg/ml	5 µg/ml	2 µg/ml	5 µg/ml	2 µg/ml	5 µg/ml	2 µg/ml	5 µg/ml
**20a**	–	+	+	++	–	+	+	++
**20b**	+	+	+	+	+	++	+	+
**20c**	–	+	+	+	–	+	+	+

Inhibition zone diameter: (–) < 11 mm [inactive]; (+) 11–14 mm [weakly active]; (++) 15–18 mm [moderately active]

**Scheme 6:**
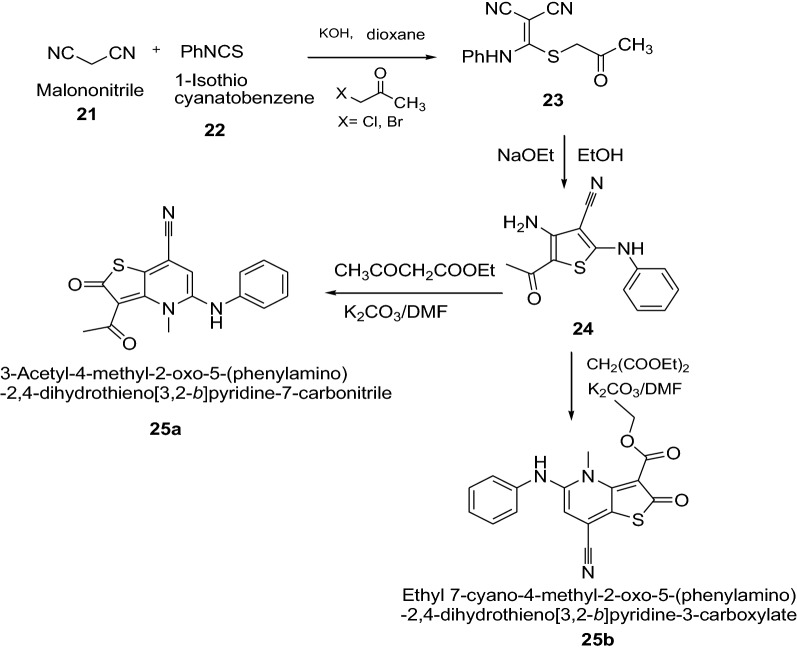
Synthesis of Ethyl 7-cyano-4-methyl-2-oxo-5-(phenylamino)-1,2-dihydrothieno[3,2-*b*]pyridine

Ahmed et al. [19] synthesized thieno[3,2-*b*]pyridine-2-one derivatives by using Scheme 6. The synthesized thienopyridines derivatives were evaluated for their in vitro antibacterial activity against two grampositive (*B. subtilis* and *S. aureus*) and two Gram-negative (*E. coli* and *S. typhi*) strains using paper disk diffusion assay method by comparing with amoxicillin (30 μg/disk) as reference antibiotic. The compounds **25a** and **25b** showed remarkable biological activity because of the substitution of the CN (at C3) either by acetyl (as in **25a**) and/or ethoxycarbonyl (as in **25b**). However, the antibacterial activity was slightly hampered by the existence of the electron withdrawing *p*-bromophenyl group at fourth position of carbon. The results of synthesized compounds presented in Table 6.

**Table 6:** The in vitro antibacterial activity of the synthesized thienopyridines derivatives

Compound	Gram-positive species		Gram-negative species	
	*B. Subtilis*	*S. aureus*	*E. coli*	*S. typhosa*
**25a**	12.5	12.5	25	50
**25b**	6.3	12.5	25	50

Minimum Inhibitory Concentrations was expressed as (µg/ml)

**Scheme 7:**
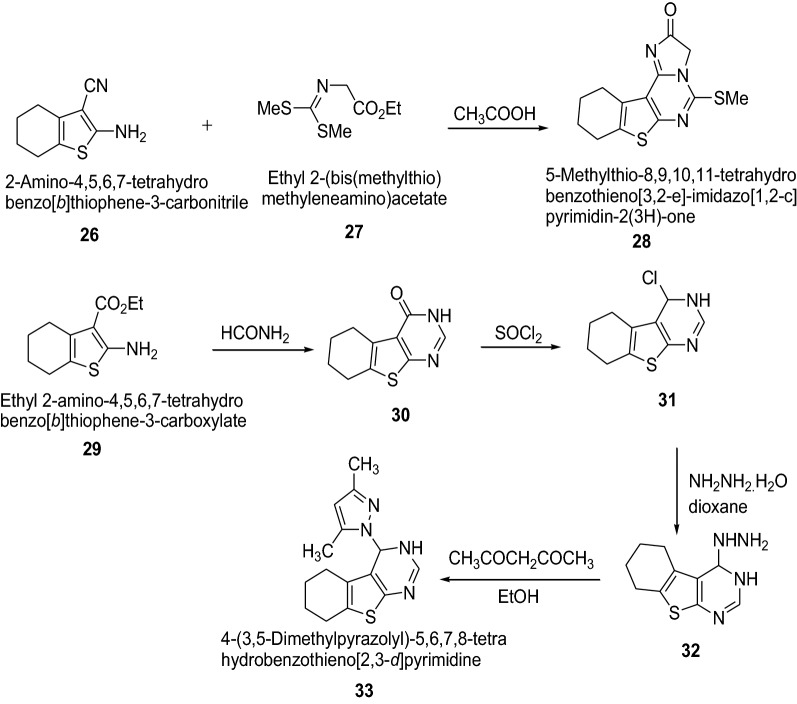
Synthesis of thienopyrimidine derivatives

Bhuiyan et al. [20] synthesized a novel class of [1,2,4]triazolo[4,3-*c*]thieno-[3,2-*e*] pyrimidine derivatives using Scheme 7 and assayed for the antibacterial activity against *B. cereus*, *S. dysenteriae* and *S. typhi* and for antifungal activity against *M. phaseolina*, *F. equiseti*, *A. alternate* and *C. corchori*. The disc diffusion method and poisoned-food techniques were used for antibacterial and antifungal activities, respectively. Among the synthesized compounds **28** and **33** resulted in wide spectrum antimicrobial activity against all the test bacteria and fungi using ampicillin and nystatin as a standard drug, respectively. Introduction of imidazo (**28**) or pyrazolo (**33**) moiety to the pyrimidine derivatives might be responsible for enhancement of antimicrobial activity of these compounds. The results of synthesized compounds are presented in Tables 7 and 8.

**Table 7:** Antibacterial activity of some synthesized compounds

Compound	Zone of inhibition (mm)		
	*B. cereus*	*S. dysenteriae*	*S. typhi*
**28**	20	13	26
**33**	31	28	29
Ampicillin	21	30	24

1 mg/ml per disc

**Table 8:** Antifungal activity of some synthesized compounds

Compound	Inhibition of mycelial growth (%)^a^			
	*M. phaseolina*	*F. equiseti*	*A. alternate*	*C. corchori*
**28**	34.5	37.5	54	50
**33**	48.3	65.6	65.4	54.5
Nystatin	71.8	44.7	51.6	40.5

^a^1 mg/ml per disc

**Scheme 8:**
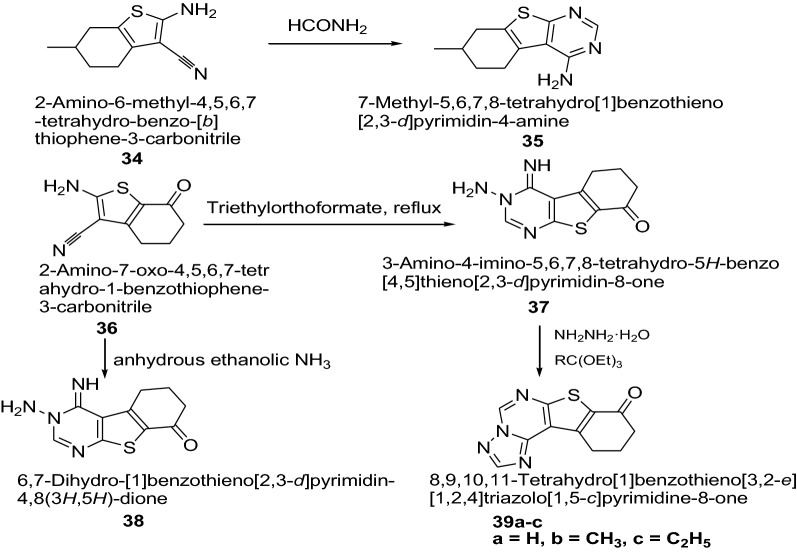
Synthesis of thienopyrimidines and triazolothienopyrimidines derivatives

Khazi et al. [21] developed some novel tricyclic thienopyrimidines and triazole fused tetracyclic thienopyrimidines derivatives by employing the Gewald reaction (Scheme 8). The synthesized compounds were evaluated against two Gram positive bacteria (*S. aureus*, *B. subtilis*), two Gram negative bacteria (*P. aeruginosa*, *E. coli*) and two yeast-like fungi *C. albicans* and *C. parapsilosis* using the broth micro dilution method. The result indicated that the compounds **35**, **37**, **39a**, **39b** and **39c** have exhibited good antibacterial activity against *B. subtilis* comparable to the standard ampicillin, while compound **38** displayed better antifungal activity against *C. albicans* comparable to the standard fluconazole. The results of synthesized compounds are presented in Table 9.

**Table 9:** Antibacterial and antifungal activities of the compounds as MIC values (μg/ml)

Compounds	*S. aureus*	*B. subtilis*	*E. coli*	*P. aeruginosa*	*C. albicans*	*C. parapsilosis*
**35**	256	11	128	256	128	64
**37**	512	256	256	256	16	128
**38**	512	11	256	256	128	256
**39a**	128	11	128	256	128	128
**39b**	256	11	128	256	256	64
**39c**	128	11	256	128	128	64
Ampicillin	4	8	4	–	–	–
Fluconazole	–	–	–	–	8	0.25

**Scheme 9:**
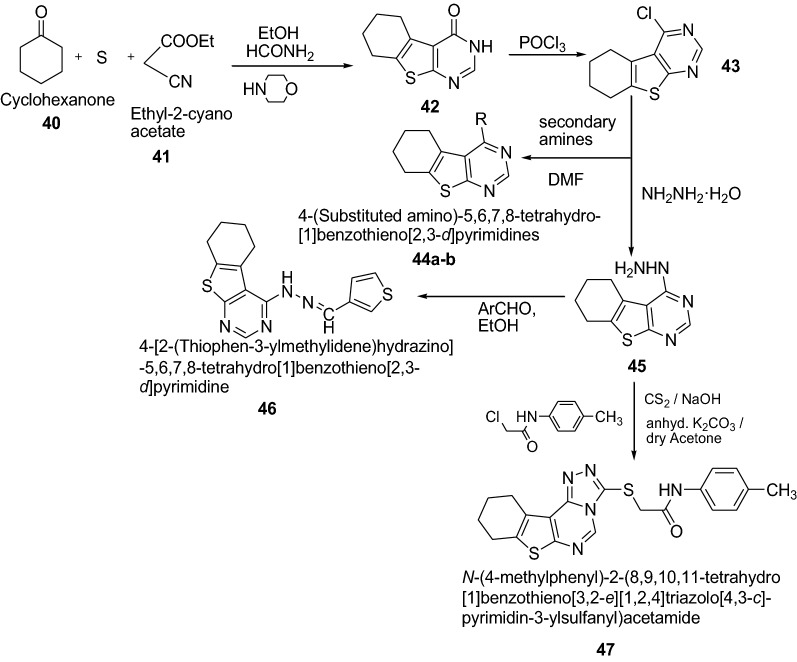
Synthesis of tetrahydrobenzothieno[2,3-*d*]pyrimidine and tetrahydrobenzothienotri azolopyrimidine

Tombary et al. [22] synthesized series of tetrahydrobenzothieno[2,3-*d*]pyrimidine and tetrahydrobenzothienotriazolopyrimidine derivatives as presented in Scheme 9 and evaluated for their antimicrobial activity using the cup diffusion technique against *S. aureus* as Gram-positive bacteria, *E. coli* and *P. aeruginosa* as Gram-negative bacteria in addition to *C. albicans* as fungi. The minimum inhibitory concentration (MIC) and minimum bactericidal concentration (MBC) for the active compounds were studied and compared with ampicillin and clotrimazole as reference antibiotics. Antimicrobial testing revealed that compounds **44a** and **47** were the most active among the tested compounds against *C. albicans* while compounds **44b** and **46** showed the highest antibacterial potency against *P. aeruginosa* among the tested compounds. The significant results of these compounds are presented in Table 10.

**Table 10:** Antibacterial and antifungal activities of synthesized compounds

Compound	The inhibition zones (IZ) in mm diameter			
	*S. aureus*	*E. coli*	*P. aeruginosa*	*C. albicans*
**44a**	–	15	16	22
**44b**	–	16	19	16
**46**	21	17	20	20
**47**	–	17	–	22
Ampicillin	25	28	32	–
Clotrimazole	–	–	–	35

(–) no inhibition zone, Minimum Inhibitory Concentrations was expressed as (µg/ml)

**Scheme 10:**
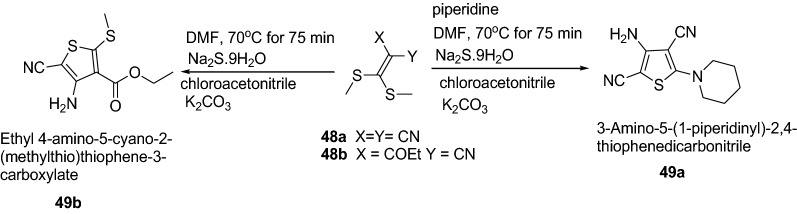
Synthesis of tetrasubstituted thiophenes derivatives

Adiwish et al. [23] synthesized tetra substituted thiophenes from ketene dithioacetals as represented in Scheme 10. The synthesized compounds **49a** and **49b** were evaluated in vitro for their antibacterial activity against Gram-positive bacteria (*S. aureus* and *B. subtilis*) and Gram-negative bacteria (*E. coli* and *K. pneumonia*) by using agar disc-diffusion technique. The result revealed that compound **49a** exhibited bigger inhibition zones compared to **49b**. The results of synthesized compounds presented in Table 11.

**Table 11:** Antibacterial activities of newly synthesized compounds

Bacteria	Inhibition zones (mm)			
	**49a**	**49b**	DMSO	Streptomycin
*B. subtilis*	–	–	–	25
*S. aureus*	9	7	–	13
*E. coli*	7	–	–	25
*K. pneumonia*	–	–	–	25

Minimum inhibitory concentrations was expressed as (µg/ml)

**Scheme 11:**
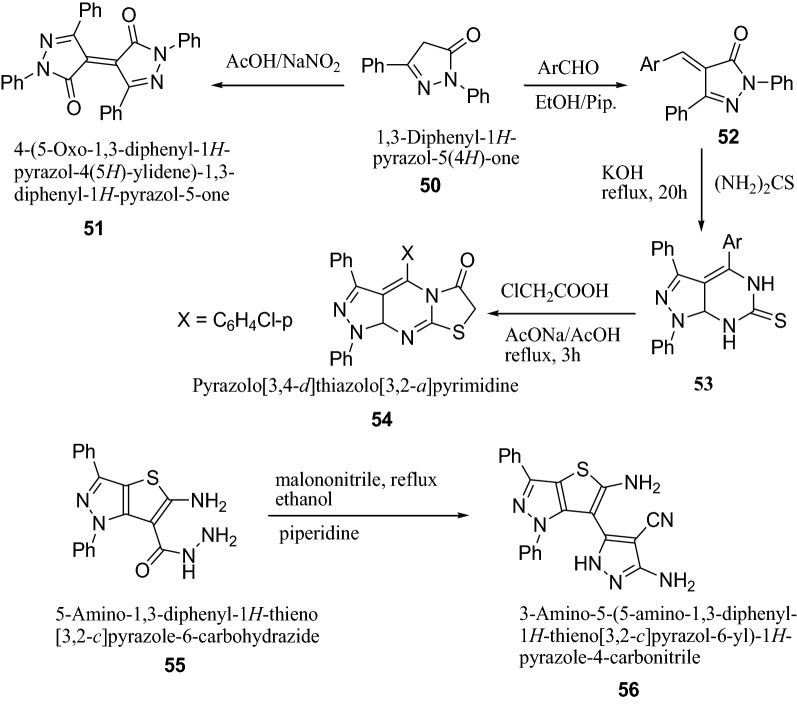
Synthesis of substituted thieno[3,2-*c*]pyrazole and pyrazolo[3′,4′:4,5]thieno[2,3-*d*]pyrimidine derivatives

Reheim et al. [24] synthesized some novel substituted thieno[3,2-*c*]pyrazole and pyrazolo[3′,4′:4,5]thieno[2,3-*d*]pyrimidine derivatives as represented in Scheme 11. The antimicrobial activity of the target synthesized compounds were screened against various microorganisms such as *E. coli*, *B. megaterium*, *B. subtilis*, *F. proliferatum*, *T. harzianum*, *A. niger* by the disc diffusion method. Antibacterial activity result indicated that among the synthesized derivatives, compounds **51**, **54** and **56** showed promising broad spectrum antibacterial activities against *E. coli*. The results of synthesized compounds presented in Table 12.

**Table 12:** Antibacterial and antifungal activities of compound as MIC values (μg/ml)

Compounds	Bacterial species			Fungal species		
	*E. coli*	*B. megaterium*	*B. subtilis*	*F. proliferatum*	*T. harzianum*	*A. niger*
**54**	15	10	20	12	12	10
**51**	20	12	20	12	15	12
**56**	15	10	15	20	10	15
Ampicillin	23	23	23	–	–	–
Clotrimazole	–	–	–	22	22	22

Inhibition zone diameter (mm)

### Anticancer activity

Cancer is among the most challenging health problems worldwide which has become a major problem for increasing mortality rate globally. Currently available treatments such as chemotherapy and radiotherapy can only provide temporary therapeutic benefits as well as being limited by a narrow therapeutic index, remarkable toxicity, and acquired resistance for most of the type of cancer. However, the research of anticancer drugs in the past several decades has shown extensive progress and has cured considerable number of patients. Still it is the extreme area of investigation due to the complex physiological changes in the cell functionality, metastasis and apoptotic mechanisms. Lots of compounds were screened for anticancer activity in the past few years because of the presence of various cell lines and screening methods. Most of the scientist has synthesized and investigated some of novel thiophene derivatives for the anticancer activity carrying the biologically active sulfonamide, isoxazole, benzothiazole, quinoline and anthracene moieties [25–27].

**Scheme 12:**
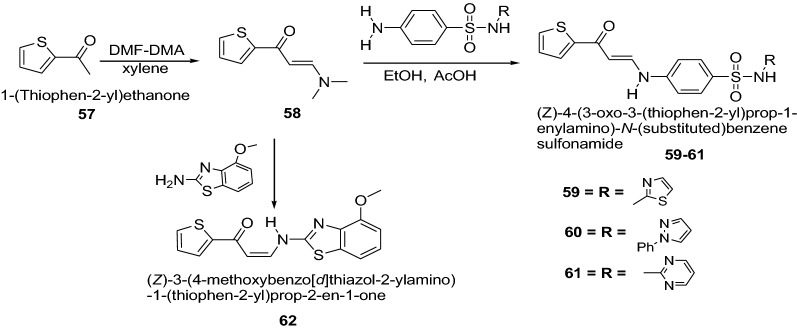
Synthesis of thiophenes having the biologically active sulfonamide (**59**–**61**) and 3-methylisoxazole 12,4-methoxybenzo[*d*]thiazole (**62**)

Ghorab et al. [28] developed a novel series of thiophenes derivatives having biologically active sulfonamide, isoxazole, benzothiazole, quinoline and anthracene moieties as presented in Scheme 12. The synthesized compounds were evaluated for in vitro anticancer activity against human breast cancer cell line (MCF7). Many of them showed cytotoxic activities compared to doxorubicin as a positive control. Among this series, (*Z*)-4-(3-oxo-3-(thiophen-2-yl)prop-1-enylamino)-*N*-(thiazol-2-yl)benzenesulfonamide (**59**), (*Z*)-4-(3-oxo-3-(thio-phen-2-yl)prop-1-enylamino)-*N*-(1-phenyl-1*H*-pyrazol-5-yl)benzenesulfonamide (**60**), (*Z*)-4-(3-oxo-3-(thiophen-2-yl)prop-1-enylamino)-*N*-(pyrimidin-2-yl)benzenesulfonamide (**61**) and (*Z*)-3-(4 methoxybenzo[*d*]thiazol-2-ylamino)-1-(thiophen-2-yl)prop-2-en-1-one (**62**) having IC_50_ values 10.25, 9.70, 9.55 and 9.39 μmol/l, respectively revealed a promising anti-breast cancer activity than that of doxorubicin with IC_50_ = 32.00 μmol/l. It was mainly due to the thiophene nucleus containing biologically active sulfathiazole **59**, sulfaphenazole **60**, sulfadiazine **61**, or benzothiazole **62** moieties. The results of synthesized compounds showed in Table 13.

**Table 13:** In vitro anticancer screening of the newly synthesized compounds against the human breast cancer cell line MCF7

Compound	Compound concentration (μmol/l)				
	Surviving fraction^a^				
	10	25	50	100	IC_50_ (μmol/l)
Doxorubicin	0.551 ± 0.026	0.480 ± 0.003	0.139 ± 0.005	0.130 ± 0.016	32.00
**59**	0.541 ± 0.003	0.323 ± 0.020	0.360 ± 0.018	0.460 ± 0.015	10.25
**60**	0.480 ± 0.010	0.327 ± 0.016	0.313 ± 0.005	0.381 ± 0.007	9.70
**61**	0.443 ± 0.017	0.251 ± 0.012	0.355 ± 0.020	0.290 ± 0.009	9.55
**62**	0.435 ± 0.009	0.233 ± 0.006	0.371 ± 0.018	0.309 ± 0.011	9.39

^a^Mean ± S.E, *n *= 3

**Scheme 13:**
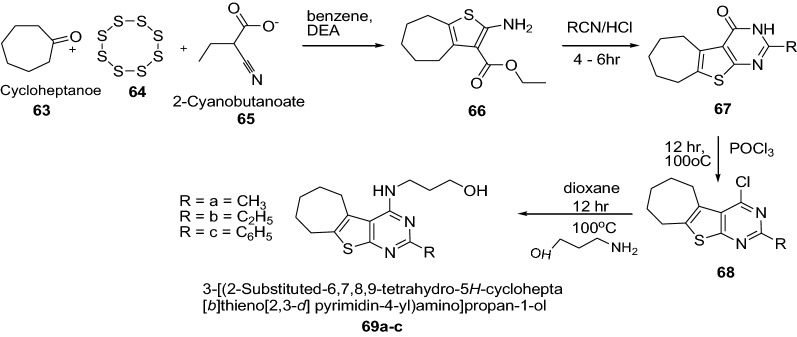
Synthesis of 3-[(2-substituted-6,7,8,9-tetrahydro-5*H*-cyclohepta[*b*]thieno[2,3-*d*]pyrimidin-4-yl)amino]propan-1-ol

Gaunda et al. [29] synthesized some new derivatives of 3-[(2-substituted-6,7,8,9-tetrahydro-5*H*-cyclohepta[*b*]thieno[2,3-*d*]pyrimidin-4-yl)amino]propan-1-ol derivatives (Scheme 13). The in vitro cytotoxicity activity of synthesized compounds were screened against both the cell lines (HC 29-Colorectal adenoma cell line and MDA 231-adenocarcinoma breast cancer cell line) by MTT assay and analyzed statistically. Among this series, the compound **69c** had shown better anticancer activity at all concentrations on both the cell lines followed by compound **69a**, **69b**. It was due to the phenyl substitution (**69c**) which has shown better anticancer activity. However, all the synthesized compounds showed considerable anticancer activity as compared to cyclophosphamide. The results of synthesized compounds presented in Table 14.

**Table 14:** Anticancer activity of the title compounds (**69a**–**69c**)

Compound code	Concentration (μmol)	Percentage inhibition of cell growth	
		HC 29-colorectal adenoma cell line (%)	MDA 231-adenocarcinoma breast cancer cell line (%)
**69a**	0.03	32.39	31.18
	0.07	31.43	29.35
	0.17	27.96	24.47
**69b**	0.03	30.73	24.94
	0.07	27.94	25.79
	0.17	25.27	26.41
**69c**	0.03	34.52	33.68
	0.07	32.83	30.72
	0.17	28.45	25.35

**Scheme 14:**
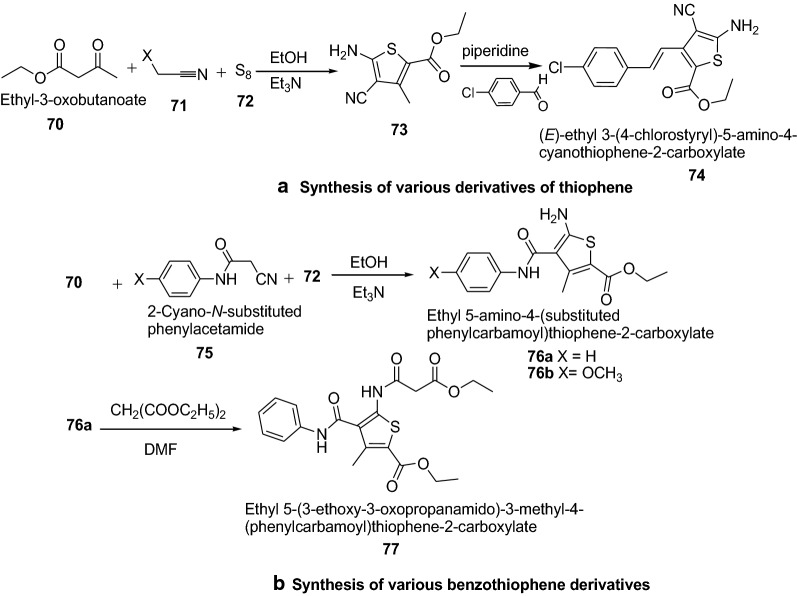
**a** Synthesis of various derivatives of thiophene, **b** Synthesis of various benzothiophene derivatives

Mohareb et al. [30] developed a convenient synthetic approach for novel thiophene and benzothiophene derivatives (Scheme 14). The in vitro cytotoxicity was screened against three tumor cell lines–MCF-7 (breast adenocarcinoma), NCI-H460 (non-small cell lung cancer) and SF-268 (CNS cancer) and a normal fibroblast human cell line (WI-38) compared to the anti-proliferative effects of the reference control doxorubicin. Among the series, ethyl-5-amino-3-(4-chlorostyryl)-4-cyanothiophene-2-carboxylate (**74**), ethyl 5-amino-4-[(4-methoxyphenyl)carbamoyl]-3-methylthiophene-2-carboxylate (**76b**) and ethyl 5-(3-ethoxy-3-oxopropanamido)-3-methyl-4-(phenylcarbamoyl)thiophene-2-carboxylate (**77**) were found to be the most active compounds against the three tumor cell lines such as MCF-7, NCI-H460 and SF-268 where as they showed low potency against the normal fibroblasts human cell line (WI-38). It was revealed that higher cytotoxicity activity of compound **74** was due to the presence of the chloro group, OCH_3_ group in compound **76b** and the presence of two ethoxy groups in compound **77**. Thus it has been shown that, in most cases, the electronegative Cl, OCH_3_ and OC_2_H_5_ hydrophobic groups in the thiophene derivatives might play a very important role in enhancing the cytotoxic effect. The results of synthesized compounds presented in Table 15.

**Table 15:** Anticancer activity of the title compounds

Compound	IC_50_ (μmol/l)^a^			
	MCF-7	NCI-H460	SF-268	WI-38
**74**	0.03 ± 0.007	0.02 ± 0.008	0.01 ± 0.004	> 100
**76b**	0.01 ± 0.006	0.03 ± 0.002	0.06 ± 0.005	> 100
**77**	0.01 ± 0.003	0.02 ± 0.001	0.01 ± 0.001	66.5 ± 12.7
DSMO	94.3 ± 6.4	96.4 ± 10.2	98.6 ± 12.2	> 100
Doxorubicin	0.0428 ± 0.0082	0.0940 ± 0.0087	0.0940 ± 0.0070	> 100

^a^Drug concentration required to inhibit tumor cell proliferation by 50% after continuous exposure of 48 h; data are expressed as mean ± SEM of three independent experiments performed in duplicates

**Scheme 15:**
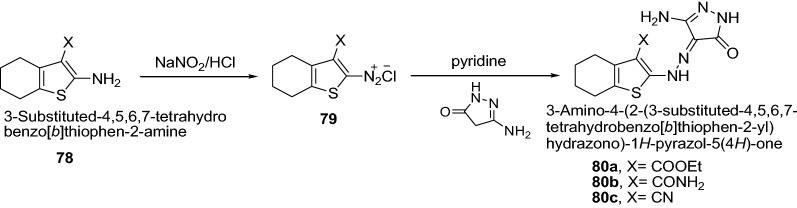
Synthesis of substituted-4-{2-[(or 3-phenyl-)4,5,6,7-tetrahydrobenzo[*b*]thiophen-2-yl]hydrazono}-1*H*-pyrazol-5(4*H*)-one derivatives

Sharkawy et al. [31] synthesized a series of thiophene incorporating pyrazolone moieties via diazo coupling of diazonium salt of 3-substituted-2-amino-4,5,6,7-tetrahydrobenzo[*b*]thiophenes with 3-methyl-1*H*-pyrazol-5(4*H*)-one, 3-methyl-1-phenyl-1*H*-pyrazol-5(4*H*)-one or 3-amino-1*H*-pyrazol-5(4*H*)-one, respectively as represented in Scheme 15. Newly synthesized derivatives were tested for cytotoxicity against the well known established model ehrlich ascites carcinoma cells (EAC) in vitro. The results showed clearly that compounds **80a**–**c** exhibited high cytotoxic activity than 5-fluorouracil which may be due to the presence of amino group in position 3 of the pyrazol-5-one moiety. Further, the order of antitumor activity of this series of synthesized compounds follows **80c** < **80b** < **80a** which may be due to replacement of CONH_2_ by CN or COOC_2_H_5_ groups of benzothiophene ring in position 3. The results of synthesized compounds showed in Table 16.

**Table 16:** Antitumor activity of synthesized compounds

Compound no	% Dead		
	100 µg/ml (%)	500 µg/ml (%)	25 µg/ml (%)
5-Fluorouracil	97.3	68	38.6
**80a**	100	98.6	94
**80b**	98.4	81	65
**80c**	98.1	79	60

The % dead refers to the % of the dead tumor cells

**Scheme 16:**
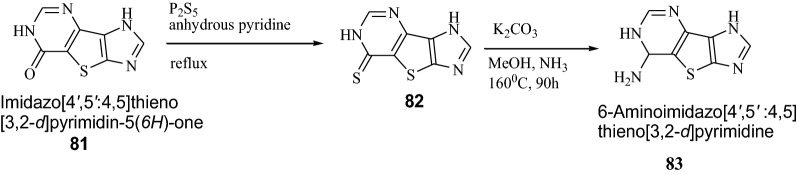
Synthesis of 6-Aminoimidazo[4′,5′:4,5]thieno[3,2-*d*]pyrimidine

Seley et al. [32] synthesized tricyclic thieno-separated purine analogues using Scheme 16. These synthesized derivatives were screened for their cytotoxic activity against HCT116 colorectal cancer cell lines. In this series, compound **83** showed potent cytotoxic activity against cancer cell lines. It was due to the coupling of compound **83** to a ribo-sugar to create the thieno-separated nucleosides may increase the growth inhibitory properties of these analogues. The results of synthesized compounds presented in Table 17.

**Table 17:** Tricyclic compound-induced inhibition of HCT116 growth

Time (h)	Compd	0.1 µM	1 µM	10 µM	100 µM
24	**83**	96.1 ± 3.8	106.6 ± 4.8	104.8 ± 3.8	82.0 ± 7.8
48	**83**	105.1 ± 3.3	98.3 ± 4.3	105.0 ± 2.1	71.5 ± 3.8^b^
72	**83**	101.3 ± 7.3	96.1 ± 6.6	93.8 ± 12.1	51.5 ± 10.7^a^

HCT116 cells were treated and growth was assessed. Data represent the average (SEM as a % of control-treated (DMSO) cells (*n *= 3–5)

^a^*p *< 0.05

^b^*p *< 0.005 when compared to control-treated cells

**Scheme 17:**
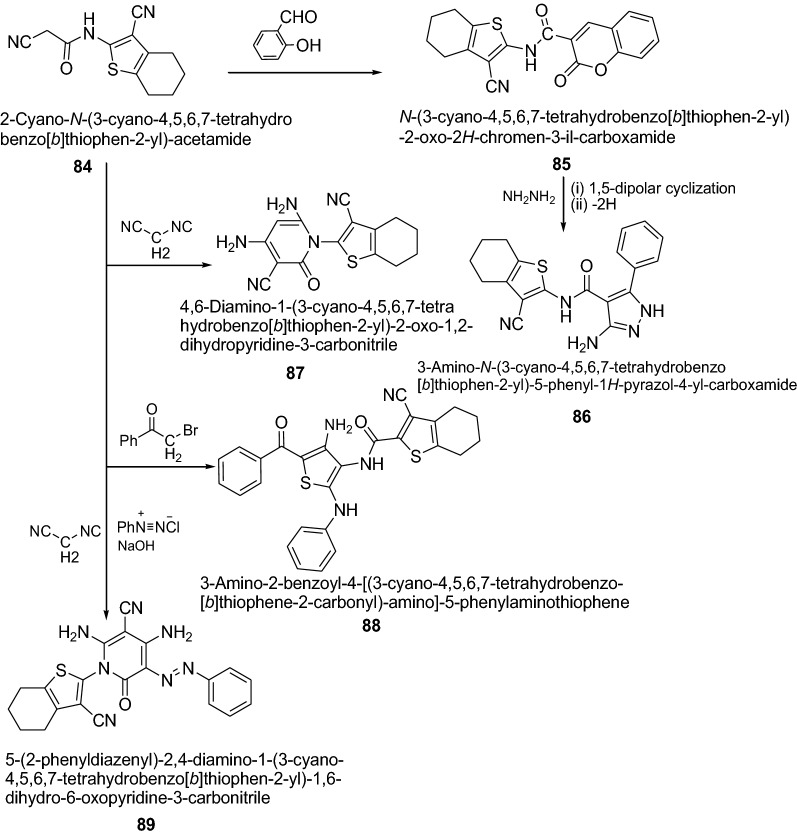
Synthesis ofsubstituted-1-(3-cyano-4,5,6,7-tetrahydrobenzo[*b*]thiophen-2-yl) derivatives

Mohareb et al. [33] synthesized novel heterocyclic compounds from 2-cyano-*N*-(3-cyano-4,5,6,7-tetrahydrobenzo[*b*]thiophen-2-yl)-acetamide as presented in Scheme 17. The tumor cell growth inhibition activities of the newly synthesized thiophene systems were assessed in vitro on three human tumor cell lines, namely, MCF-7 (breast adenocarcinoma), NCI-H460 (non-small cell lung cancer), and SF-268 (CNS cancer) after a continuous exposure of 48 h. The results were compared to the antiproliferative effects of the reference control doxorubicin. In this series, compounds **89**, **86**, **88**, **85**, and **87** showed significant activity on the three tumor cell lines tested. The results of synthesized compounds showed in Table 18.

**Table 18:** Antiproliferative activity GI50 (μM) of the synthesized compounds

Compound	GI50 (μM)^a^		
	MCF-7	NCI-H460	SF-268
**85**	10.8 ± 0.6	16.5 ± 0.8	16.7 ± 1.6
**86**	2.5 ± 0.5	10.4 ± 0.6	8.0 ± 0.4
**87**	16.7 ± 1.6	10.8 ± 0.6	16.5 ± 0.8
**88**	11.8 ± 0.6	14.5 ± 0.8	16.7 ± 1.6
**89**	2.0 ± 0.4	8.3 ± 0.8	4.0 ± 0.8
Doxorubicin	0.0428 ± 0.0082	0.0940 ± 0.0087	0.0940 ± 0.0070

^a^Drug concentration required to inhibit tumor cell proliferation by 50% after continuous exposure of 48 h. Doxorubicin was used as positive control

### Antioxidant activities

**Scheme 18:**
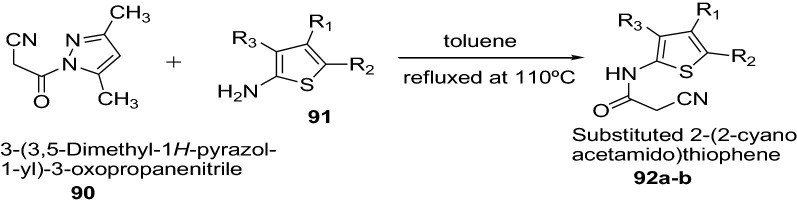
Synthesis of substituted 2-aminothiophene

Madhavi et al. [34] developed a novel class of substituted 2-(2-cyanoacetamido)thiophenes by cyanoacetylation of substituted 2-aminothiophene by using an effective cyanoacetylating agent, 1-cyanoacetyl-3,5-dimethylpyrazole as presented in Scheme 18. All the synthesized compounds were evaluated for in vitro antioxidant activity by scavenging 1,1-diphenyl-2-picrylhydrazyl (DPPH) and nitric oxide free radicals at 100 μM concentration. Among these evaluated compounds, 2-(2-cyanoacetamido)-4,5-dimethylthiophene-3-carboxamide (Compound **92a**) was found to possess highest anti-oxidant activity in both models of free radical scavenging. However in case of assay with nitric oxide free radical scavenging, the highest activity was exhibited by 2-(2-cyanoacetamido)-4,5-dimethylthiophene-3-carboxamide (Compound **92a**, 56.9%) and 2-(2-cyanoacetamido)-4,5,6,7-tetrahydrobenzo[*b*]thiophene-3-carboxamide (Compound **92b**, 55.5%). The greater activity of these compounds were attributed due to the polar nature of carboxamide or nitrile group at 3rd position on thiophene ring. The results of synthesized compounds presented in Tables 19 and 20.

**Table 19:** Reduction of DPPH by substituted 2-(2-cyanoacetamido) thiophenes

Compound	R_1_, R_2_	R_3_	% Inhibition at 100 μM
**92a**	–CH_3_	–CONH_2_	52.4
Ascorbic acid			64.7

**Table 20:** Effect of substituted 2-(2-cyanoacetamido)thiophenes on scavenging of nitric oxide

Compound	R_1_, R_2_	R_3_	% Inhibition at 100 μM
**92a**	–CH_3_	–CONH_2_	56.9
**92b**	–(CH_2_)_4_–	–CONH_2_	55.5

### Anti-inflammatory activity

**Scheme 19:**
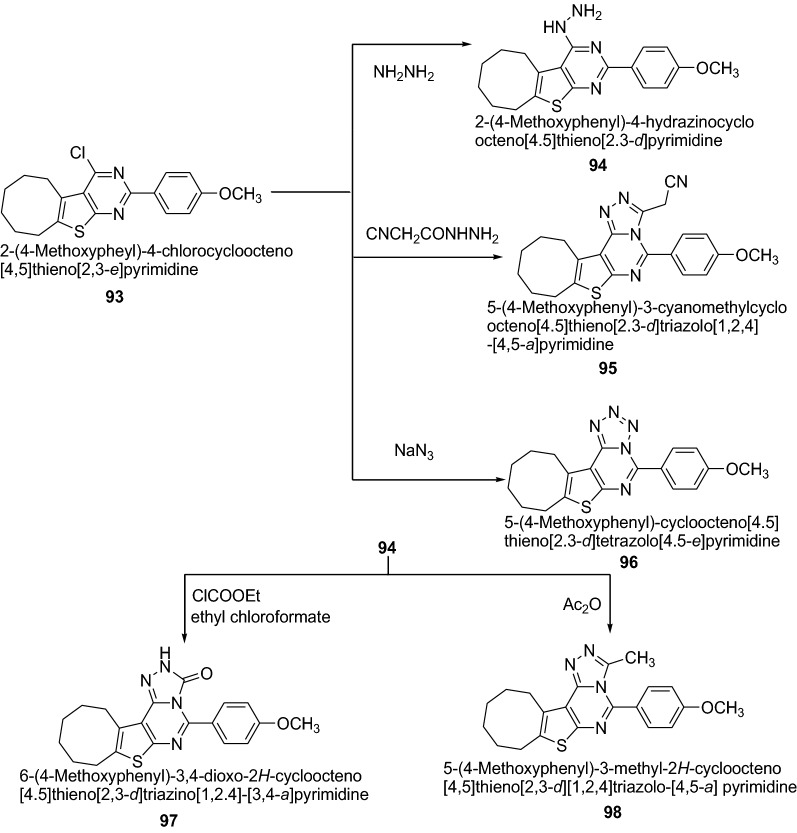
Synthesis of thienotriazolopyrimidine derivatives

Bahashwan et al. [35] synthesized new series of fused triazolo- and tetrazolopyrimidine derivatives (Scheme 19) and their anti-inflammatory activity was evaluated. Newly synthesized thienopyrimidine derivatives were screened for anti-inflammatory activity (percent inhibition of edema obtained by the reference drug and tested compounds, respectively) in comparison to that of indomethacin. Among the series, compounds **94**, **95**, **96**, **97** and **98** possess strong anti-inflammatory activity. The high anti-inflammatory activity was mainly due to the presence of electron-donating moieties which increase the pharmacological activity. The order of anti-inflammatory properties with the substitution of electron–donating group in pyrimidine derivatives follows as: hydrazine > methyl > cyanomethyl > tetrazine > amide as exhibited in compounds **94** > **98** > **95** > **96** > **97**, respectively. The results of synthesized compounds presented in Table 21.

**Table 21:** Anti-inflammatory activity of the synthesized compounds

Compounds	Edema inhibition (means ± E.M)^a,b^ (%)		
	1 h	2 h	3 h
**94**	42.3 ± 1.1	49.2 ± 1.2	57.1 ± 1.4
**95**	37.2 ± 1.3	46.3 ± 1.5	54.4 ± 1.1
**96**	34.5 ± 1.2	35.1 ± 1.5	48.2 ± 1.2
**97**	31.2 ± 1.2	35.1 ± 1.5	40.2 ± 1.6
**98**	39.1 ± 1.5	48.4 ± 1.2	55.6 ± 1.1
Indomethacin	44.7 ± 1.2	52.4 ± 1.2	61.2 ± 1.3

^a^Dose 5 mg/kg b.m (p.o.)

^b^ n = 6

**Scheme 20:**
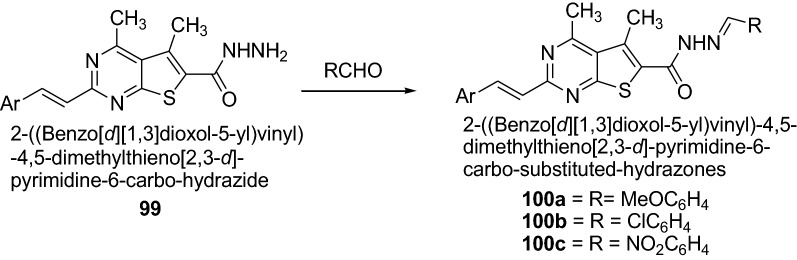
Synthesis of 2-((Benzo[*d*] [1, 3] dioxol-5-yl)vinyl)-4,5-dimethylthieno[2,3-*d*]-pyrimidine-6-carbohydrazones derivatives

Ouf et al. [36] synthesized hydrazones derivatives which shows significant anti-inflammatory activities as presented in Scheme 20. The synthesized compounds were screened against the standard drug flurbiprofen. Among the synthesized hydrazones, the substituted 4-methoxy- **100a**, 4-chloro- **100b** and 4-nitro-derivatives **100c** have anti-inflammatory activities higher than that of hydrazone with an unsubstituted benzaldehyde group against the standard drug flurbiprofen. Thus, the lipophilicity plays an important role for the potent anti-inflammatory activity. The results of synthesized compounds presented in Table 22.

**Table 22:** Anti-inflammatory activity of some synthesized compounds (% reduction in edema induced by yeast)

Compound	Post treatment 3 h = %	Post treatment 6 h = %
**100a**	26.6	34.4
**100b**	17.2	30.0
**100c**	24.2	34.2

**Scheme 21:**
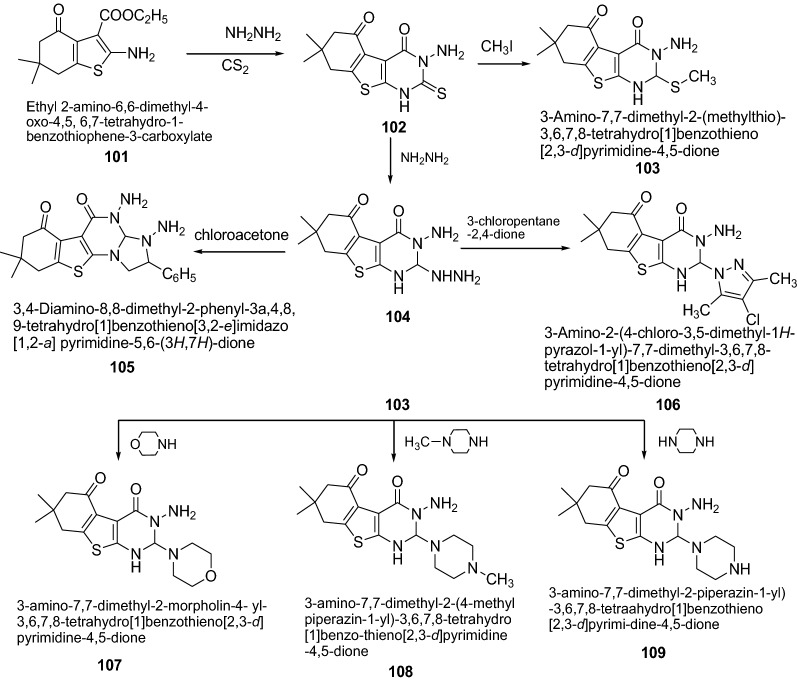
Synthesis of phenylpyrazolothinopyrimidine, morphonyl and piperazinylthinopyrimidine derivatives

Hafez et al. [37] synthesized some of the novel benzothino-pyrimidine derivatives (Scheme 21) which showed considerable potent anti-inflammatory activity. The anti-inflammatory activity of the newly synthesized compounds were evaluated by applying carrageenan-induced paw edema bioassay in rats using indomethacin as a reference standard. Compounds **105**, **106**, **107**, **108** and **109** caused significant decreases in paw edema after 2, 3, 4 h after drug administration. Thus, it can be concluded that spirobenzothienopyrimidine moiety, phenylpyrazolothinopyrimidine, morphonyl and piperazinylthinopyrimidine ring systems are important for anti-inflammatory activity. The results of synthesized compounds presented in Table 23.

**Table 23:** Anti-inflammatory effect of synthesized compounds

Compd. no.	Oedema volume			
	1 h	2 h	3 h	4 h
**105**	49.4 ± 7.1	71.8 ± 6.7^a^	76.4 ± 4.8^a^	82.2 ± 5.2
**106**	44.2 ± 5.1	59.6 ± 4.7^a^	67.8 ± 3.3^a^	81.9 ± 3.2
**107**	61.0 ± 6.6	66.6 ± 5.9^a^	68.6 ± 7.0^a^	72.4 ± 7.4
**108**	66.4 ± 7.5	78.2 ± 3.5^a^	81.3 ± 3.3	87.1 ± 2.1
**109**	56.2 ± 9.9	65.1 ± 7.5^a^	55.9 ± 10.6^a^	54.7 ± 7.2^a^
Indomethacin	49.8 ± 5.3	42.9 ± 5.1^a^	45.9 ± 4.6^a^	46.9 ± 5.8^a^

^a^p < 0.05: Statistically significant from the control using one way ANOVA (Two-sided Dunnett as Post Hoc test)

### Antiurease activity

**Scheme 22:**
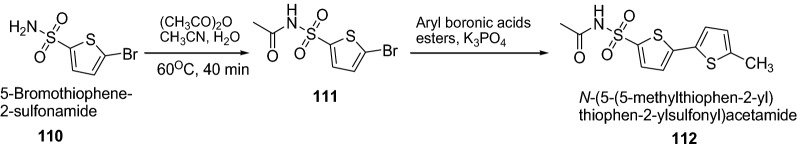
Synthesis of *N*-(5-(5-methylthiophen-2-yl)thiophen-2 ylsulfonyl)acetamide

Rasool et al. [38] synthesized variety of novel 5-aryl thiophenes derivatives containing sulphonylacetamide (sulfacetamide) using Scheme 22. The synthesized compounds were screened for their anti-urease activities by taking thiourea as standard drug. Among all the synthesized derivatives, compound **112**, *N*-((5′-methyl-[2,2′-bithiophen]-5-yl)sulfonyl)acetamide, showed excellent urease inhibition activity at 40 µg/ml and 80 µg/ml concentrations where the percentage inhibition values were found to be 92.12 ± 0.21 and 94.66 ± 0.11, respectively with an IC_50_ value ~ 17.1 ± 0.15 µg/ml. It is further concluded that the urease inhibitory activity of compound might be due to the presence of the electronic and steric effects of functional groups. The results of synthesized compounds are presented in Table 24.

**Table 24:** Urease inhibition studies of 5-arylthiophene-2-sulfonylacetamides

Compound	Percentage activity at 15 µg/ml	Percentage activity at 40 µg/ml	Percentage activity at 80 µg/ml	IC_50_ µg/ml
**112**	42.44 ± 0.11	92.12 ± 0.21	94.66 ± 0.11	17.1 ± 0.15
Standard	47.1 ± 0.31	65 ± 0.01	–	23.3 ± 0.21

Standard = Thiourea

### Anticonvulsant activity

**Scheme 23:**
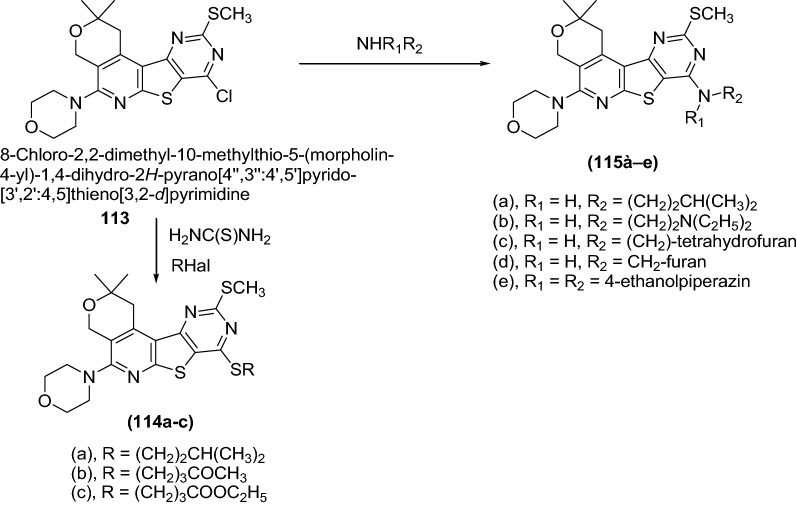
Synthesis of 2,4-Disubstituted pyrano[4′,3′:4,5]pyrido[2,3-*b*]thieno[3,2-*d*]pyrimidine derivatives

Dashyan et al. [39] synthesized 2,4-disubstituted pyr ano[4′′,3′′:4′,5′]pyrido[3′,2′:4,5]thieno[3,2-*d*]pyrimidines derivatives by using Scheme 23. The synthesized compounds were screened for the anticonvulsant activity of by taking the comparator drug, diazepam which was performed using male albino mice weighing 18–24 g (200 animals) and rats (Wistar) weighing 120–140 g (40 animals of both sexes).

The anticonvulsant activity of the compounds was assessed by the prevention of clonic twitches and the clonic component of convulsions caused by subcutaneous administration of 90 mg/kg metrazol in mice. When studying anticonvulsant activity, it was found that the compounds (**114a**, **b**, **c**) and (**115a**, **b**, **c**, **d**, **e**) caused a marked protective anticonvulsive effect, which developed in mice starting with a dose of 25 mg/kg, while statistically calculated dose (ED_50_) ranged from 23 to 56 mg/kg (Table 25).

**Table 25:** Activity against metrazol convulsions for the compounds (**114a**, **b**, **c**), (**115a**, **b**, **c**, **d**, **e**), and diazepam

Compound	Activity against metrazol convulsions* (ED50, mg/kg)
**114a**	56.0 (36.0–100.8)
**114b**	40.0 (23.5–68.0)
**114c**	23.0 (15.9–33.1)
**115a**	40.0 (23.5–68.0)
**115b**	56.0 (36.0–100.8)
**115c**	34.0 (25.3–45.7)
**115d**	40.0 (23.5–68.0)
**115e**	56.0 (36.0–100.8)
Diazepam	0.51 (0.39–0.69)

* Probability levels at *p *= 0.05 are indicated in brackets

### Antithrombotic activity

**Scheme 24:**
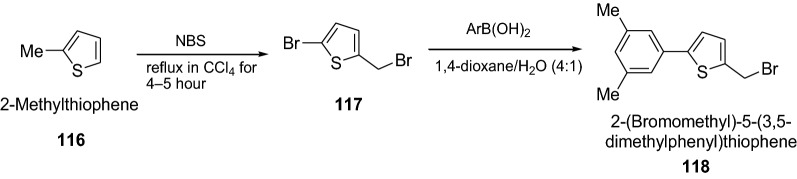
Synthesis of 2-(Bromomethyl)-5-(3,5-dimethylphenyl)thiophene

Jubair et al. [40] synthesized novel series of 2-(bromomethyl)-5-aryl-thiophenes derivatives via Suzuki cross-coupling reactions of various aryl boronic acids with 2-bromo-5 (bromomethyl)thiophene as given in Scheme 24. The synthesized compounds were screened for their antithrombolytic activity. All the Compounds (100 μL) having concentration of 1 mg/ml were added to the micro-centrifuge tubes containing venous blood, and incubated at 37 °C for 45 min. Streptokinase was used as standard clot lysis agent and water as negative control for this assay. Among all the synthesized compounds, **118** showed potent clot lysis (31.5%). However, the results were significant p < 0.05, when compared with streptokinase. Clot lysis activity results are presented in Table 26.

**Table 26:** Percentage efficiency of Clot lysis of synthetic compounds

Compounds	Clot lysis %
**118**	31.5 ± 0.45
Water	0.43 ± 0.005
Streptokinase	87.2 ± 0.95

The results are average ± S.D of triplicate experiments p < 0.05

## Conclusion

The analytical and other informational data, available in literature so far, have reveals that thiophene and its derivatives represent an important class of compounds in the medicinal field with various therapeutic potentials, i.e., antimalarial, antimicrobial, antimycobacterial, antidepressant, anticonvulsant, antiviral, anticancer, antihypertensive, anti-inflammatory and antioxidant. Appraisal of literature reports reveals that thiophene moiety have hiked a great deal of interests of medicinal chemist and biochemist to plan, organize and implement new approaches towards discovery of novel drugs.

This particular review article, established the fact that thiophene derivatives could be a rich source of potential entities in search of new generation of biologically active compounds and be worthwhile to explore the possibility in this area by fusing differently substituted moieties which may result in better pharmacological activities. Thus the quest to explore many more modifications on thiophene moiety needs to be continued.
